# Molecular analysis of non-O1/non-O139 *Vibrio cholerae* isolated from hospitalised patients in China

**DOI:** 10.1186/1471-2180-13-52

**Published:** 2013-03-04

**Authors:** Yun Luo, Julian Ye, Dazhi Jin, Gangqiang Ding, Zheng Zhang, Lingling Mei, Sophie Octavia, Ruiting Lan

**Affiliations:** 1 Zhejiang Provincial Center for Disease Control and Prevention, Hangzhou, Zhejiang, China; 2 School of Biotechnology and Biomolecular Sciences, University of New South Wales, Sydney, NSW, 2052, Australia

**Keywords:** *Vibrio cholerae*, Non-O1/non-O139 serogroups, Pulsed-field gel electrophoresis, Multilocus sequence typing, Antibiotic resistance, Type III secretion system

## Abstract

**Background:**

Cholera is still a significant public health issue in developing countries. The aetiological agent is *Vibrio cholerae* and only two serogroups, O1 and O139, are known to cause pandemic or epidemic cholera. In contrast, non-O1/non-O139 *V. cholerae* has only been reported to cause sporadic cholera-like illness and localised outbreaks. The aim of this study was to determine the genetic diversity of non-O1/non-O139 *V. cholerae* isolates from hospitalised diarrhoeal patients in Zhejiang Province, China.

**Results:**

In an active surveillance of enteric pathogens in hospitalised diarrhoeal patients, nine non-O1/non-O139 *V. cholerae* isolates were identified from 746 diarrhoeal stool samples at a rate of 1.2%. These isolates and an additional 31 isolates from sporadic cases and three outbreaks were analysed using pulsed-field gel electrophoresis (PFGE) and multilocus sequence typing (MLST). PFGE divided the isolates into 25 PFGE types while MLST divided them into 15 sequence types (STs). A single ST, ST80, was predominant which persisted over several years in different cities and caused two outbreaks in recent years. Antibiotic resistance varied with the majority of the isolates resistant to sulphamethoxazole/trimethoprim and nearly all isolates either resistant or intermediate to erythromycin and rifampicin. None of the isolates carried the cholera toxin genes or toxin co-regulated pilus genes but the majority carried a type III secretion system as the key virulence factor.

**Conclusions:**

Non-O1/non-O139 *V. cholerae* is an important contributor to diarrhoeal infections in China. Resistance to commonly used antibiotics limits treatment options. Continuous surveillance of non-O1/non-O139 *V. cholerae* is important for control and prevention of diarrhoeal infections.

## Background

Cholera is an acute diarrhoeal disease caused by toxigenic *Vibrio cholerae.* The two most important serogroups are O1 and O139, which can cause periodic outbreaks reaching epidemic or pandemic proportions [1]. However, non-O1/non-O139 serogroups have been linked with cholera-like-illness sporadically [2-6]. Symptoms may range from mild gastroenteritis to violent diarrhoea, similar to those elicited by the O1 toxigenic strains [7]. However, patients generally suffer a less severe form of the disease than those infected by O1 toxigenic strains [8-10]. Non-O1/non-O139 *V. cholerae* strains have also caused localised outbreaks in many countries, including India and Thailand [3,11-15]. More recently, an O75 *V. cholerae* outbreak associated with the consumption of oysters was reported in the USA [5,6].

Non-O1/non-O139 *V. cholerae* strains are frequently isolated from the environment, particularly from seafood and aquatic sources [11,16,17]. Non-O1/non-O139 *V. cholerae* strains are highly heterogeneous with considerable serological diversity and vary in virulence properties. The presence of virulence genes amongst some environmental strains is significant, and environmental strains constitute a reservoir of potential pathogenic strains to human diarrhoeal infections [18-21]. Some non-O1/non-O139 strains carry key virulence genes, such as cholera toxin (CT) and toxin co-regulated pili (TCP), which are usually carried by epidemic strains [22]. Some may also carry other virulence factors such as the repeat-like toxin (RtxA) - a cytotoxin and the heat-stable enterotoxin (NAG-ST) [4,18,22-26]. A novel type III secretion system (T3SS) was found in some non-O1/non-O139 strains and appears to be an important virulence factor [27-29]. The T3SS translocates a number of T3SS effectors into the host cell which interfere with host cell signalling [27,28]. Shin *et al.*[29] showed that T3SS is an essential virulence factor for the non-O1/non-O139 strain AM-19226.

In this study, 40 non-O1/non-O139 *V. cholerae* isolates from hospitalised diarrhoeal patients in Zhejiang Province, China were analysed using multilocus sequence typing (MLST) and pulsed-field gel electrophoresis (PFGE) to determine their overall genetic relatedness. The presence of key virulence genes including enterotoxins, TCP and T3SS was also analysed.

## Results and discussion

### Isolation of non-O1/non-O139 *V. cholerae* isolates from diarrhoeal patients in Zhejiang, China

**Figure 1:**
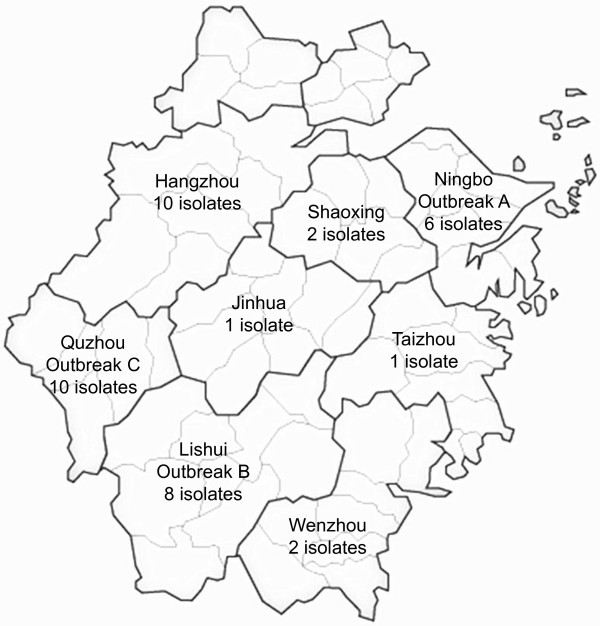
**Geographical map of Zhejiang Province, China.** Cities are demarcated with dark solid lines. City names together with number of non-O1/non-O139 *Vibrio cholerae* isolates from a given city is shown.

A total of 40 non-O1/non-O139 *V. cholerae* isolates was retrieved from different cities in Zhejiang Province, China, over a period of six years from 2005 to 2011 (Figure 1, Table 1). Nine isolates were from sporadic cases from seven cities, while 22 isolates were obtained from three outbreaks in three different cities: outbreak A in Ningbo in 2005, outbreak B in Lishui in 2006 and outbreak C in Quzhou in 2011. The three outbreaks were notified as food poisoning events and were investigated. Outbreak A involved 20 cases with symptoms ranging from cholera-like diarrhoea to mild diarrhoea and was initially suspected to be a cholera outbreak. Non-O1/non-O139 *V. cholerae* was isolated from nine patients. The outbreak occurred in a factory canteen and the food source of the outbreak could not be identified. Outbreak B involved eight cases, all having cholera-like symptoms. Non-O1/non-O139 *V. cholerae* was isolated from all but one patient. The source of the outbreak was traced to cross contamination of a cold dish from raw cuttlefish. Outbreak C occurred in a family function involving 12 cases with non-O1/non-O139 *V. cholerae* isolated from nine cases. The source of the outbreak was shrimp.

**Table 1:** **Details of *Vibrio cholerae *strains analysed in this study**

**Strain**	**Place**^**†**^	**Source**	**Year**	**Pulse type**	***ctxAB***	***toxR***	***hlyA***	tcpA^*^	***zot***	**NAG-ST**	**T3SS**	**T3SS**	***adk***	***gyrB***	***mdh***	***metE***	***pntA***	***purM***	***pyrC***	**Sequence type**
											**( *vcsC2 *)**	**( *vcsV2 *)**								
N11192	HZ	sporadic	2011	24	-	+	+	-	-	-	-	-	39	45	14	67	50	1	42	93
N10001	HZ	surveillance	2010	13	-	+	+	-	-	-	+	+	2	44	11	64	6	8	10	94
N10002	HZ	surveillance	2010	N/A^‡^	-	+	+	-	-	-	+	+	2	23	45	48	47	35	5	89
N10003	HZ	surveillance	2010	14	-	+	+	-	-	-	+	+	2	5	45	56	6	36	51	80
N10004	HZ	surveillance	2010	15	-	+	+	-	-	-	+	+	2	38	14	56	6	36	51	83
N10005	HZ	surveillance	2010	16	-	+	+	-	-	-	+	+	2	5	45	56	6	36	51	80
N10006	HZ	surveillance	2010	17	-	+	+	-	-	-	+	+	2	5	45	56	6	36	51	80
N10007	HZ	surveillance	2010	18	-	+	+	-	-	-	-	+	19	46	14	22	48	1	52	86
N10008	HZ	surveillance	2010	19	-	+	+	-	-	-	-	-	38	47	44	65	49	1	53	91
N10009	HZ	surveillance	2010	20	-	+	+	-	-	-	+	+	2	5	45	56	6	36	51	80
N11191	JH	sporadic	2011	23	-	+	+	-	-	-	-	-	24	36	1	50	30	18	45	81
N743	LS	outbreak B	2006	8	-	-	+	-	-	-	+	+	2	5	45	56	6	36	51	80
N744	LS	outbreak B	2006	9	-	+	+	-	-	-	+	+	2	5	45	56	6	36	51	80
N745	LS	outbreak B	2006	10	-	+	+	-	-	-	+	-	2	5	45	56	6	36	51	80
N746	LS	outbreak B	2006	11	-	+	+	-	-	-	+	+	2	5	45	56	6	36	51	80
N747	LS	outbreak B	2006	9	-	+	+	-	-	-	+	+	2	5	45	56	6	36	51	80
N748	LS	outbreak B	2006	9	-	+	+	-	-	-	+	+	2	5	45	56	6	36	51	80
N749	LS	outbreak B	2006	9	-	+	+	-	-	-	+	+	2	5	45	56	6	36	51	80
N750	LS	sporadic	2007	12	-	+	+	-	-	-	+	+	11	27	11	63	19	1	4	88
N733	NB	outbreak A	2005	2	-	+	+	-	-	-	+	+	2	5	29	19	6	36	51	82
N734	NB	outbreak A	2005	2	-	+	+	-	-	-	+	+	2	5	29	19	6	36	51	82
N735	NB	outbreak A	2005	3	-	+	+	-	-	-	+	+	2	5	29	19	6	36	51	82
N737	NB	outbreak A	2005	2	-	+	+	-	-	-	+	+	2	5	29	19	6	36	51	82
N738	NB	outbreak A	2005	2	-	+	+	-	-	-	+	+	2	5	29	19	6	36	51	82
N739	NB	outbreak A	2005	4	-	+	+	-	-	-	+	+	2	5	29	19	6	36	51	82
N11193	QZ	outbreak C	2011	17	-	+	+	-	-	-	+	+	2	5	45	56	6	36	51	80
N11194	QZ	outbreak C	2011	25	-	+	+	-	-	-	-	-	27	5	14	22	30	18	45	92
N11196	QZ	outbreak C	2011	17	-	+	+	-	-	-	+	+	2	5	45	56	6	36	51	80
N11198	QZ	outbreak C	2011	17	-	+	+	-	-	-	+	+	2	5	45	56	6	36	51	80
N11199	QZ	outbreak C	2011	17	-	+	+	-	-	-	+	+	2	5	45	56	6	36	51	80
N11200	QZ	outbreak C	2011	17	-	+	+	-	-	-	+	+	2	5	45	56	6	36	51	80
N11201	QZ	outbreak C	2011	25	-	+	+	-	-	-	-	-	27	5	14	22	30	18	45	92
N11202	QZ	outbreak C	2011	17	-	+	+	-	-	-	+	+	2	5	45	56	6	36	51	80
N11203	QZ	outbreak C	2011	17	-	+	+	-	-	-	+	+	2	5	45	56	6	36	51	80
N740	QZ	sporadic	2006	6	-	+	+	-	-	-	-	+	26	5	46	50	31	14	45	90
N11041	SX	sporadic	2011	21	-	+	+	-	-	-	+	+	19	1	14	66	48	1	52	87
N11072	SX	sporadic	2011	22	-	+	+	-	-	-	+	+	3	41	4	48	47	35	5	84
N742	TZ	sporadic	2006	7	-	+	+	-	-	-	+	+	11	27	11	63	19	1	4	88
N732	WZ	sporadic	2005	1	-	+	+	-	-	-	+	+	3	23	15	48	47	35	5	85
N736	WZ	sporadic	2005	5	-	+	+	-	-	-	+	+	3	23	15	48	47	35	5	85

^†^ Place corresponds to different cities in Zhejiang province: *HZ* - Hangzhou; *JH* - Jinhua; *LS* - Lishui; *NB* - Ningbo; *QZ* - Quzhou; *SX* - Shaoxing; *TZ* - Taizhou; and *WZ* – Wenzhou.

^‡^ The isolate was unable to be typed by PFGE.

^*^Two primer pairs of *tcpA* (see Table 2) were used. Both were negative.

Nine other non-O1/non-O139 *V. cholerae* isolates were obtained during an active surveillance of enteric bacterial pathogens conducted by Zhejiang Provincial CDC in two Provincial hospitals in Hangzhou between May and December in 2010. These nine cases of non-O1/non-O139 *V. cholerae* infections were identified from a total of 746 diarrhoeal stool samples screened. All samples were screened for *Salmonella, Shigella, Campylobacter, Yersinia enterocolitica,* pathogenic *Vibrio* spp., pathogenic *E. coli, Aeromonas hydrophila, Plesiomonas shigelloides,* rotavirus, enteric adenovirus, norovirus, sapovirus, and astrovirus. There were no other enteric pathogens isolated from these nine cases. This data gave a non-O1/non-O139 *V. cholerae* infection rate of 1.2 per 100 diarrhoeal patients. Thus, non-O1/non-O139 *V. cholerae* is an important pathogen in this population and has been neglected as a pathogen generally.

The prevalence of non-O1/non-O139 *V. cholerae* in clinical samples varied in other countries. In Thailand, the proportion of non-O1/non-O139 *V. cholerae* isolated from diarrhoeal patients was between 1.0 and 1.3% [3], which is comparable to our study. In Italy, two non-O1/non-O139 *V. cholerae* infections (3.4%) were identified among 58 hospitalized patients with acute diarrhoea and both were associated with seafood consumption [30]. In cholera endemic regions, isolation of non-O1/non-O139 *V. cholerae* seems to be higher. In a 2003 survey in Kolkata, India, non-O1/non-O139 *V. cholerae* constituted 27.4% of the total *V. cholerae* isolations from hospitalised patients with acute diarrhoea [16], although estimates based on the number of diarrhoeal cases were not available.

### Molecular typing of non-O1/non-O139 *V. cholerae* isolates

**Figure 2:**
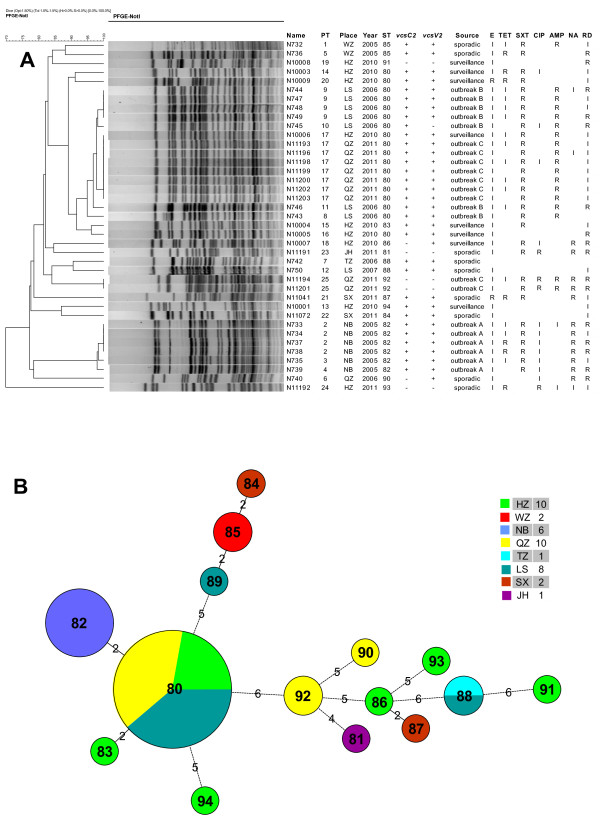
**Relationships of the non-O1/non-O139 *Vibrio cholerae *isolates. A**. Dendrogram analysis generated using the unweighted pair group method with arithmetic based on pulsed field gel electrophoresis (PFGE) patterns. Place corresponds to different cities in Zhejiang province: HZ - Hangzhou; JH - Jinhua; LS - Lishui; NB - Ningbo; QZ - Quzhou; SX - Shaoxing; TZ - Taizhou; and WZ – Wenzhou. The classification of the PFGE type (PT), sequence type (ST); presence (+) or absence (−) of the two T3SS genes (*vcsC2* and *vcsV2*); and resistance (R) or intermediate (I) to antibiotics (E - erythromycin, TET - tetracycline, SXT - sulphamethoxazole/trimethoprim, CIP – ciprofloxacin, AMP – ampicillin, NA - nalidixic acid and RD – rifampicin) is shown. **B**. Minimum spanning tree based on MLST data. The number in the circle indicates the ST and the size of the circle corresponds the total number of isolates belonging to that ST. Different localities are indicated in colour and specified in the colour legend together with the total number of isolates from each city in brackets. City name abbreviations are the same as in A above. The number of allelic difference between STs is indicated on the branches. Nodes were connected by a dashed line if the difference is more than two alleles.

In order to determine the genetic and epidemiological relatedness among the isolates, we first performed PFGE analysis using the PulseNet standardised PFGE protocol for *V. cholerae*. PFGE is the gold standard of epidemiological typing as it offers high discriminatory power [31] and is routinely used for epidemiological typing of food-borne pathogens by the Zhejiang Provincial Center for Disease Control and Prevention. Thirty nine of the 40 isolates were typed using PFGE and were divided into 25 PFGE types (PTs) (Figure 2A). Of the six outbreak A isolates, four belonged to the same PFGE pattern (PT2), while the other two had two different patterns (PT3 and PT4) with only one band difference to PT2. Four outbreak B isolates had the same PFGE pattern (PT9) and three others had a unique pattern (PT8, PT10 and PT11). PT9 and PT10 were very similar to each other while PT11 and PT8 differed by three and four bands from PT9 respectively. The nine outbreak C isolates were separated into two distinctive patterns (PT17 with seven isolates and PT25 with two isolates). The similarity in PFGE patterns among the isolates was estimated using dice coefficient and was represented by a dendrogram in Figure 2A. The dendrogram showed that outbreak C was most likely caused by two different strains since PT17 and PT25 were well separated in the dendrogram. Interestingly, one isolate (N10006) obtained in the 2010 active surveillance in Hangzhou shared the same PFGE pattern (PT17) with seven outbreak C isolates from Quzhou. It seems that the PT17 strain causing the 2011 outbreak in Quzhou has been circulating in the neighbouring Hangzhou city a year earlier.

All ST80 outbreak C isolates (PT17) were grouped together but were placed within outbreak B PTs and were closest to PT9 and PT10 (Figure 2A). It should be noted that PT17 looked nearly identical to PT9 in Figure 2A. However, closer examination of the PFGE patterns showed that the two bands in PT17 clearly were not identical to those in PT9. Since the two outbreaks were separated by time and locality, it is interesting to note such a close relationship of the isolates, which also shows that epidemiological information must be considered in addition to PFGE patterns in detecting outbreaks.

We further used multilocus sequence typing (MLST) to determine the relationships of and genetic heterogeneity among the isolates. Seven housekeeping genes (*adk*, *gyrB*, *metE*, *mdh*, *pntA*, *purM* and *pyrC*) selected based on a previous study [32] were used for the MLST (Octavia *et al.* manuscript in preparation). MLST divided the 40 isolates into 15 sequence types (STs) (Figure 2B). ST80 was predominant which consisted of 18 isolates. eBURST [33] analysis showed none of the STs formed a clonal complex. A minimum spanning tree was constructed (Figure 2B) which showed three sets of closely related STs: ST82, ST83 and ST80; ST84, ST85 and ST89; and ST86 and ST87, all differing by two genes. Two STs (ST80 and ST88) were isolated over two or more years and from different cities, suggesting that these two STs had a wide geographical distribution. For the three outbreaks, outbreak A was caused by ST82 while outbreaks B and C were caused by ST80. However, the ST80 isolates from outbreaks B and C can be separated by one band difference by PFGE. Additionally, two of the nine outbreak C isolates belonged to ST92. Therefore, outbreak C was caused by two STs and possibly due to contamination of the source (shrimp) by two different strains. There was also heterogeneity in isolates from the same city. The nine isolates from the 2010 active surveillance in Hangzhou were separated into six STs. Thus, our MLST analysis showed that these non-O1/non-O139 isolates were genetically diverse and some strains such as those belonging to ST80 can predominate across the regions.

We compared the relationships of isolates based on MLST (Figure 2B) with those based on PFGE. For the five STs (ST80, ST82, ST85, ST88 and ST92) with two or more isolates, each individual ST is associated with distinct PFGE nodes with all isolates of the same ST contained within the same node (Figure 2A). Additionally, two isolates of different STs, N10004 of ST83 and N10005 of ST80 were grouped together by PFGE with a three-band difference and a 95% similarity (Figure 2A). This was consistent with the MLST relationship as ST83 was linked with ST80 with a two-allele difference (Figure 2B). The two alleles differed between ST83 and ST80 were *gyrB* and *mdh* with 5 bp and 4 bp differences, respectively. The differences in these genes may be due to recombination as *V. cholerae* undergoes recombination quite frequently [32]. Therefore, relationships of isolates with high similarity in PFGE patterns are consistent between PFGE and MLST.

In contrast, the relationships of isolates with less similar PFGE patterns were inconsistent with those based on MLST. For example, the ST86 isolate N10007 was grouped together with the ST81 isolate N11191 by PFGE, while by MLST ST81 and ST86 were not linked together on the MST (Figure 2B). These two isolates differed substantially in their banding patterns (Figure 2B) and also differed in all seven alleles by MLST. Similarly the grouping together of ST84 and ST94 by PFGE was also inconsistent with their relationship based on MLST (Figure 2B).

As measured by the index of diversity (D), the discriminatory power of PFGE (D = 0.945) was clearly higher than MLST (D = 0.781) for characterisation of non-O1/non-O139 *V. cholerae*. PFGE further divided isolates within an ST for all STs except ST92 in which there were only two isolates and both were from the same outbreak.

### Antibiotic resistance patterns amongst non-O1/non-O139 *V. cholerae* isolates

Antimicrobial susceptibility testing was carried out using disk diffusion assay for 13 antibiotics and all or nearly all isolates were susceptible to cephalothin, cefotaxime, gentamicin, amikacin, doxycycline and norfloxacin. Resistance to other antibiotics varied with 80% of the isolates resistant to sulphamethoxazole/trimethoprim (SXT), 47.5% to ampicillin, 42.5% to rifampicin, 30% to nalidixic acid, 15% to tetracycline, 5% to ciprofloxacin and 5% to erythromycin. Additionally, for rifampicin, erythromycin and tetracycline, the majority or nearly all of the remaining isolates were intermediate to the respective antibiotics (Figure 2A). Isolates obtained from the same outbreak may also vary in antibiotic resistance. However, most of these variations were due to intermediate resistance (Figure 2A). The use of antimicrobial agents is generally regarded as an effective method to reduce the duration and symptoms of diarrhoea. Tetracycline, erythromycin, SXT and ciprofloxacin have all been generally considered as the drug of choice for the treatment of cholera. However, the resistance profiles indicate that these antibiotics will not be or less effective for treating non-O1/non-O139 *V. cholerae* infections.

Antibiotic resistance profiles were also correlated with PFGE or MLST relationships. All ST82 isolates and all except one ST80 isolate were resistant to SXT. The only SXT susceptible ST80 isolate was grouped away from the other ST80 isolates. All ST80 isolates associated with outbreaks (either outbreak B or outbreak C) were resistant to ampicillin. Nalidixic acid resistance also has a restricted distribution. With the exception of the nalidixic acid resistant ST90 isolate (N740) and the nalidixic acid resistant ST87 isolate (N11041) which are unrelated, nalidixic acid resistance was present only in the two ST92 outbreak C isolates, all ST82 outbreak A isolates and the two related ST86 and ST81 isolates. The two ST92 isolates were the most drug resistant and shared the same resistance profile with resistance or intermediate to six antibiotics (erythromycin, SXT, ciprofloxacin, ampicillin, nalidixic acid and rifampicin). The ST86 and ST81 isolates (N10007 and N11191, respectively) grouped together by PFGE shared a similar resistance profile with resistance or intermediate to five antibiotics (erythromycin, SXT, ciprofloxacin, nalidixic acid and rifampicin).

The distribution of SXT resistance on the tree (Figure 2A) revealed an interesting evolutionary history. SXT resistance in *V. cholerae* is carried by a conjugative, self-transmissible and integrative element (SXT element) that also provides resistance to chloramphenicol and streptomycin [18,34,35]. The wide distribution of SXT resistance along the tree suggests that the SXT element is widespread, although previous studies mostly analysed *V. cholerae* O1 and O139 toxigenic strains for the presence of SXT element [35-37]. Interestingly, one of the ST80 isolates which was obtained from the 2010 active surveillance was susceptible to SXT and presumably has lost the SXT element since every other isolate in the large PFGE clusters was resistant to SXT. In contrast, the four SXT susceptible isolates (two ST88 isolates, one ST84 isolate and one ST94 isolate) were grouped together as two pairs of isolates on different branches of the tree and are likely to have not gained the SXT element. Resistance to the other antibiotics may be due to chromosomal mutations, plasmids or other mobile elements [38] and are more difficult to make any evolutionary inference of the observed resistance patterns.

### Detection and distribution of virulence factors genes

PCR assays (Table 2) were used for the detection of the *ctxAB*[39], *tcpA*[40], *zot*[41], NAG-ST [16], T3SS (*vcsC2* and *vcsV2*) [16,28], *ompW*[42], *toxR*[42] and *hlyA* genes [43]. All isolates were positive for *V. cholerae* specific gene *ompW* by PCR, but were negative for *ctxAB*, *zot*, *tcpA* and *NAG-ST.* All isolates were positive for *toxR* (Table 1), except for N743 which was *toxR* negative. Interestingly, N743 also differed from other ST80 isolates in its PFGE pattern. *toxR* codes for the transcriptional regulatory protein ToxR [44] and is expected to be present in all *V. cholerae* isolates. Negative PCR amplification of *toxR* from N743 may be due to sequence divergence in primer binding regions*.* Similarly, all isolates were positive for the haemolysin gene *hlyA* (Table 1). In contrast, the absence of *ctxAB*, *zot*, *tcpA* and *NAG-ST* suggests that these non-O1/non-O139 isolates caused diarrhoea by a different mechanism from that used by toxigenic *V. cholerae* O1 and O139.

**Table 2:** PCR primers used in this study

**Gene target**	**Primer sequence (5’-3’)**		**Probe**	Ta*	**Amplicon size (bp)**	**Reference**
	**Forward**	**Reverse**				
*ompW*	TCCTCAACGCTTCTGTGTGGTAT	ATTGATTTCAACATCCGTGGATT	FAM-TGAAACAACGGCAACCTACAAAGCAGG-BHQ1	55	92	This study
*hlyA*	AGTGGTCAACCGATGCGATT	TTCAGGATCTGCGCTTTATTGTT	ROX-CCCAAGATTATCGCTTCGTGTTTAACGCA- BHQ2	47-55	76	This study
*toxR*	GATTCGACAAAGTCCCCACAA	TCGGGCGATCAATTGGTAA	HEX-CGTCAAAACGGTTCCGAAACGCG-BHQ1	47-55	66	This study
*ctxAB*	CTCAGACGGGATTTGTTAGGCACG	TCTATCTCTGTAGCCCCTATTACG	-	55	303	[39]
*tcpA (1)*^*#*^	GTGACTGAAAGTCATCTCTTC	AATCCGACACCTTGTTGGTA	-	55	1248	[40]
*tcpA (2)*^*#*^	ATATGCAATTATTAAAACAGC	TTATTATTACCCGTTGTCGG	-	55	1052	[40]
*ace*	AGAGCGCTGCATTTATCCTTATTG	AACTCGGTCTCGGCCTCTCGTATC	-	55	655	[41]
*zot*	GCTATCGATATGCTGTCTCCTCAA	AAAGCCGACCAATACAAAAACCAA	-	55	1000	[41]
T3SS *(vcsC2)*	GGAAAGATCTATGCGTCGACGTTACCGATGCTATGGGT	CATATGGAATTCCCGGGATCCATGCTCT AGAAGTCGGTTGTTTCGGTAA	-	47-60	535	[16]
T3SS *(vcsV2)*	ATGCAGATCTTTTGGCTCACTTGATGGG	ATGCGTCGACGCCACATCATTGCTTGCT	-	47-55	742	[16]
NAG-ST	CCTATTCATTAGCATAATG	CCAAAGCAAGCTGGATTGC	-	47-55	215	[16]

^*^Ta – Annealing temperature.

^#^ Two primer pairs of *tcpA* primers were used. These two primer pairs have been used previously to amplify divergent *tcpA* alleles [24].

Recent reports suggest that T3SS is present in some non-O1/non-O139 isolates and plays an important role in virulence [16,28]. We tested for the presence of T3SS using two T3SS genes (*vcsC2* and *vcsV2*). Thirty two carried both genes and presumably have a functional T3SS while five were negative for both genes. Three isolates were negative for one of the genes, two isolates negative for *vcsC2* and one isolate negative for *vcsV2*. The primer binding regions in the genes of these isolates may be divergent leading to non-amplification, but it is also possible that the genes are deleted. It seemed that the pathogenicity of the majority of the isolates was due to the presence of the T3SS since 35 isolates possessed one or both T3SS genes (87.5%),which is different from that reported in Bangladesh (38.9%) [45] and in India (31.5%) [16]. The varying presence of virulence factors among different non-O1/non-O139 strains may be associated with their ability to cause disease. Further studies are warranted.

## Conclusion

Our study is the first report which showed that non-O1/non-O139 *V. cholerae* was an important pathogen in China, causing diarrhoeal infections with an isolation rate of 1.2%. MLST revealed that a single ST, ST80, was predominant in Zhejiang Province. ST80 persisted over several years and appeared in different cities. It caused two outbreaks in recent years. Since the majority of the isolates were positive for T3SS but negative for any other virulence factors tested, the T3SS was likely to be the key virulence factor for these isolates. Resistance to commonly used antibiotics limits choice of drugs for treating non-O1/non-O139 *V. cholerae* infections. Our study highlights that non-O1/non-O139 *V. cholerae* has been neglected as an important cause of diarrhoea in China and may be the same in other developing countries. Close monitoring of non-O1/non-O139 *V. cholerae* capable of causing outbreaks in China is necessary to reduce the health burden of diarrhoeal infections caused by this pathogen.

## Methods

### Bacterial isolates

Faecal samples from sporadic and outbreak cases were collected by local hospitals as part of standard patients care over a five year period from diarrhoeal patients at local hospitals in Zhejiang Province, China, and were sent to Zhejiang Provincial CDC laboratory for isolation of *V. cholerae*. Potential *V. cholerae* isolates from the faecal samples were grown onto No. 4 Agar (1% sodium citrate, 0.5% pig gall powder, 0.003% rivano powder, 0.2% sodium sulphite, 0.1% sodium lauryl sulphate, 0.001% potassium tellurite, and 500 μg/L gentamicin). All retrieved isolates were serologically tested for agglutination of O1 or O139 antisera (Denka Seiken, Japan) and all were shown to be negative.

*V. cholerae* isolates were also obtained from an active surveillance program of enteric bacterial pathogens which was coordinated by Zhejiang Provincial CDC and was conducted in two Provincial hospitals in Hangzhou between May and December in 2010. Faecal specimens were obtained with written informed consent of the patients and with the approval of the Zhejiang Provincial CDC ethics committee, according to the medical research regulations of Ministry of Health, China.

### Molecular techniques

DNA was prepared using DNeasy Blood & Tissue kit (QIAGEN, Inc., Valencia, CA). Seven housekeeping genes (*adk*, *gyrB*, *metE*, *mdh*, *pntA*, *purM* and *pyrC*) selected based on a previous study [32] were used for the MLST (Octavia *et al.* manuscript in preparation). The amplified products were sequenced commercially by Beijing Genomics Institute. PFGE was performed according to the US CDC PulseNet standardised PFGE protocol for *V. cholerae*[31]. Simplex PCR assays (Table 2) were used for the detection of *ctxAB*[39], *tcpA*[40], *zot*[41], NAG-ST [16], T3SS (*vcsC2* and *vcsV2*) [16,28], and performed in a Mastercycler (Eppendorf, Hamburg, Germany). The reactions were carried out as follows: 5 min at 94°C; followed by 30 cycles of 30 s at 94°C, 30 s at the annealing temperature specified in Table 2, and 30 s at 72°C; followed by a final 5 min at 72°C. For detection of *ompW*[42], *toxR*[42] and *hlyA*[43] genes, new primer pairs (Table 2) were designed to be used in a multiplex real time PCR assay. The reaction was performed in an ABI7500 fast real-time PCR system (Applied Biosystems, CA, USA). The cycling conditions were as follows: 2 min at 95°C, followed by 40 cycles of 15 s at 95°C, and 45 s at the annealing temperature specified in Table 2. Isolate N10002 was typed by MLST and PCR but not typed by PFGE nor tested for antibiotic sensitivity as only DNA was available.

### Bioinformatics

Sequence alignments were done using ClustalW [46]. The PFGE dendrogram was constructed using the unweighted pair group method with arithmetic mean algorithm and Dice coefficient of two patterns at 0.5% pattern optimisation and 1.5% band position tolerance, available from Bionumerics (Applied Math). Note that one band in PT17 (band 16 from higher molecular weight end, Figure 2A) was recognised as two bands by the software to which manual correction was applied to become one band as this affected the placement of PT17. Sequence types were numbered from ST80 onwards. ST1 to ST79 were pre-assigned to isolates of another study (Octavia *et al.* manuscript in preparation). eBURST [33] was used to identify clonal complexes which are defined using the difference of one out of the seven genes typed. Minimum spanning tree using the allelic difference between isolates of the seven housekeeping genes was constructed using Bionumerics (Applied Math). The Simpson’s index of diversity (D value) [47] was calculated using an in-house program, MLEECOMP package [48].

### Antibiotic resistance

Antimicrobial susceptibility testing for 13 antibiotics including amikacin, ampicillin, cephalothin, cefotaxime, ciprofloxacin, doxycycline, erythromycin, gentamicin, nalidixic acid, norfloxacin, rifampicin, SXT and tetracycline, was carried out using disk diffusion assay according to the protocol of the Clinical and Laboratory Standards Institute [49]. Antibiotic discs were purchased from Oxoid (Hampshire, UK). Results were analysed using WHONET 5.4 software (WHO Collaborating Centre for the Surveillance of Antibiotics Resistance, Geneva, Switzerland). Isolates were classified as susceptible, intermediate or resistant based on the guidelines for each antibiotic.

### GenBank accession numbers

The sequences obtained in this study have been submitted to GenBank with accession numbers JX905826-JX05848.

## Competing interests

The authors declare that they have no competing interests.

## Authors’ contributions

Experimental work and data collection were carried out by YL, JY, DJ, GD, ZZ, LM. YL, RL and SO contributed to data analysis and interpretation. The study was conceived and designed by YL and RL. The manuscript was drafted by YL, RL and SO. All authors have read and approved the final manuscript.
